# Endothelial Functioning and Hemodynamic Parameters in Rats with Subclinical Hypothyroid and the Effects of Thyroxine Replacement

**DOI:** 10.1371/journal.pone.0131776

**Published:** 2015-07-09

**Authors:** Cuixia Gao, Tingting Li, Jing Liu, Qian Guo, Limin Tian

**Affiliations:** 1 Departments of Ultrasonic Diagnosis, Gansu Provincial Hospital, Lanzhou, Gansu, China; 2 Department of Clinical Medicine, Gansu University of Chinese Medicine, Lanzhou, Gansu, China; 3 Departments of Endocrinology, Gansu Provincial Hospital, Lanzhou, Gansu, China; Universidade Federal do Rio de Janeiro, BRAZIL

## Abstract

**Objective:**

Subclinical hypothyroidism (SCH) and its associations with atherosclerosis (AS) and cardiovascular disease remain controversial. The purpose of our study was to observe changes in endothelial functioning and hemodynamics in rats with SCH and to determine whether L-thyroxine (L-T4) administration affects these changes.

**Methods:**

In total, sixty male Wistar rats were randomly divided into the following three groups with 20 rats each: control euthyroid rats, SCH rats and SCH rats that had been treated with thyroxine (SCH+T4). The SCH rats were induced by administration of 10 mg.kg^-1^.d^-1^ methimazole (MMI) once daily by gavage for 3 months. The SCH+T4 rats were administered the same dose of MMI for three months in addition to 2 μg.kg^-1^.d^-1^ L-T4 once daily by gavage after 45 days of MMI administration. The control rats received physiological saline via gavage.

**Results:**

The SCH group had significantly higher thyroid-stimulating hormone (TSH), total cholesterol (TC), low-density lipoprotein cholesterol (LDL-C), and endothelin (ET) levels and a lower nitric oxide (NO) level than the control and SCH+T4 groups. The tail and carotid artery blood pressures, left ventricular systolic pressure, heart rate and aorta ventralis blood flow were significantly lower in the SCH group than in the control and SCH+T4 groups. ACH treatment caused concentration-dependent relaxation, which was reduced in the SCH arteries compared with the control and SCH+T4 arteries. Histopathological examination revealed the absence of pathological changes in the SCH rat arteries.

**Conclusions:**

These findings demonstrate that L-T4 treatment ameliorates endothelial dysfunction and hemodynamic changes in SCH rats.

## Introduction

Subclinical hypothyroidism (SCH), the mildest form of hypothyroidism, is defined by an increased concentration of thyroid-stimulating hormone (TSH) in the presence of a normal thyroid hormone concentration[1]. A growing body of evidence suggests that SCH has important clinical impacts on atherosclerosis (AS)[2, 3] and coronary endothelial dysfunction[4], leading to an increased risk of cardiovascular (CV) disease[5]. Moreover, previous studies have indicated that an abnormal TSH level is a risk factor for heart disease[6, 7]. TSH affects the heart rate[8], ventricular functioning and coronary artery disease risk [9].

The endothelium plays a major role in modulating vascular function and structure through the production of vasodilator and vasoconstrictor substances[10]. Endothelial dysfunction is related to impaired nitric oxide (NO) bioavailability, which can be interrelated with decreased antioxidant defense activity and increased superoxide anion generation[11, 12]. Decreased NO generation of circulating concentrations is an early physiological event in AS and can be used as a prognostic factor for CV disease[13]. Previous studies have demonstrated that endothelial dysfunction has a negative prognostic impact on the long-term outcome of coronary heart disease[14, 15]. However, the crucial mechanism of this endothelial dysfunction remains unclear.

The cardiovascular system is one of the most important targets of thyroid hormone, but the underlying cellular mechanisms are complex. Cardiac muscle function changes are generally related to myocyte calcium flow and decreased expression of various contractile proteins[16, 17]. Thyroid hormone alters calcium uptake by the sarcoplasmic reticulum to stimulate plasma membrane Ca-ATPase activity and to increase voltage-dependent channels in animal ventricular cells[17, 18]. Previous studies have demonstrated the presence of typical cardiovascular alterations in SCH[19]. However, data on the associations between SCH, CHD and mortality are conflicting[20–22] because studies that have evaluated heart function parameters have demonstrated normal results[23]. Although the use of L-T4 therapy for SCH could improve CV risk[24–28], controversy exists regarding the favorable influence of this therapy on CV risk factors.

The aim of the present study was to evaluate the effects of SCH on parameters of cardiac and endothelial functioning, especially cardiac functioning, as measured by a Millar pressure-volume conductance catheter system. Early identification of SCH may allow for its early treatment and thereby favorably affect cardiovascular morbidity and mortality. We have designed the present study to determine the effects of L-T4 treatment on endothelial and cardiac functioning.

## Materials and Methods

### Animal model

In total, sixty male *Wistar rats* weighing 140–160 g were purchased from the College of Gansu Traditional Chinese Medicine Experimental Animal Center for use in the present experiments. All of the rats were maintained under the same environment, including the same temperature and humidity, and were provided free access to food and water. The animals were randomly divided into the following three groups: control euthyroid rats (C), subclinical hypothyroid rats (SCH), and subclinical hypothyroid rats treated with L-T4 (SCH+T4) (n = 20 in each group). SCH was induced in the rats by administration of 10 mg.kg-1.d-1 [29–32] methimazole (MMI) once daily by gavage for 3 months. SCH+T4 rats were prepared using the same MMI administration schedule for three months and administration of 2 μg.kg-1.d-1 L-T4[33–35] once daily by gavage after 45 days of MMI administration. References 29–32 established hypothyroid rats, but we established SCH rats. During our experiment, we used a dosage of MMI five-fold less than their studies. Therefore, we also tested T4 and TSH levels to verify the success of the induction of SCH in our experimental groups. After 45 days, we detected increased levels of TSH and normal levels of T4 in the rats, so the chosen dose of MMI was effective for inducing SCH in the experimental groups. The control rats received physiological saline via gavage. During the 3-month period, body weight was measured daily, and the serum thyroid hormone level was measured every two weeks to ensure that the serum thyroxine (T4) level was not decreased [36, 37]. Because triiodothyronin (T3) and thyroxine (T4) levels are within normal range in SCH rats, monitoring T4 levels can reflect thyroid hormone levels.

### Drugs

All of the drugs used in the experiments were obtained commercially from Sigma and were freshly prepared in Krebs-bicarbonate solution. MMI and L-T4 were dissolved in physiological saline.

### Hemodynamic parameters

Tail blood pressure was measured in the morning in a quiet environment. Tail systolic blood pressure (SBP), mean blood pressure (MBP), and diastolic blood pressure (DBP) were recorded using tail-cuff plethysmography in unanesthetized rats (LE5001-Pressure Meter; Letica, Barcelona, Spain).

The rats were anesthetized with pentobarbital sodium (65 mg/kg i.p.). Aorta ventralis blood flow was recorded using a LPRB4291 transducer connected to a two-channel Letigraph 2000 recorder (Dutch MR, Ithaca, New York).

Body temperature was maintained with a heating pad. Arterial blood pressure was measured by cannulating the right carotid artery with a micro-tip pressure-volume conductance catheter (Millar, SPR-838 NR). After further advancing the catheter into the left ventricle, the ventricular pressures, heart rate and volumes were registered. The catheter position was optimized for maximal stroke volume (SV). After cannulation, a 10–15 min equilibration period occurred before performing the measurements. All of the measurements were conducted in spontaneously breathing rats without mechanical ventilation. All of the signals were recorded and analyzed using a PowerLab system and software (AD Instruments, Dunedin, New Zealand)[38].

Subsequently, blood samples collected from the arteria femoralis were used to determine the plasma thyroid hormone (FT3 and FT4), TSH, lipid (TG, TC, HDL-C, and LDL-C), NO and ET levels.

### Biochemical measurements

FT4, FT3, TSH and ET serum concentrations were measured using ELISA kits (Yuanye Biotechnology, Shanghai, China). Serum total cholesterol, high-density lipoprotein cholesterol (HDL-C), low-density lipoprotein cholesterol (LDL-C) and triglyceride levels were measured via enzymatic and colorimetric methods with assay kits (Sinopharm Chemical Reagent Beijing Co., Ltd, China). The serum NO level was measured using nitrate reductase and the Griess reaction in frozen samples (Bioengineering Institute, Nanjing, China). Absorbance was continuously measured at 540 nm, and data was collected every 10 s using EZ Chrom software (Scientific Software, San Ramon, CA).

### Vessel preparation and endothelium-dependent relaxation studies

The experiments were performed using abdominal aortic rings. The abdominal aorta was excised and placed into cold modified Krebs-Henseleit buffer composed of the following (in mM): NaCl, 113; KC1, 4.8; CaCl2, 2.5; MgSO4, 1.2; KH2PO4, 1.2; NaHC03, 25.0; edetate calcium disodium, 0.026; and glucose, 11.1 (control solution). Blood vessels were cleaned of adherent connective tissue and cut into rings (3–5 mm long). The rings were suspended between two stirrups in organ chambers filled with 25 ml Krebs-Ringer bicarbonate solution (37°C) that had been aerated with 95% O2 and 5% CO2. The rings were stretched to a tension of 1 g and were allowed to equilibrate for 60 min before contraction with KCl (20 mM) to assess tissue viability. Increases in tension were detected using Grass FT03 force transducers and were recorded with a Graphtech Linear recorder (FW33701) via an amplifier. The rings were constricted with norepinephrine (3×10–7 M) (NE) until steady-state constriction was observed. Dose-response experiments were performed with increasing concentrations of acetylcholine (ACH) from 10-9 to 10-4 M, and the resulting vasorelaxation was recorded[39].

### Histopathology of the arcus aortae

Parts of the descending aorta were fixed in paraformaldehyde phosphate buffer solution and routinely processed to prepare paraffin sections. At least 4–8 sections were prepared from each specimen. The sections were separated by 20 μm to obtain approximately random sections for morphometric measurements. The sections were stained with hematoxylin and eosin (HE) for microscopic examination[40].

### Ethics statement

This study was performed in strict accordance with the recommendations of the Guide for the Care and Use of Laboratory Animals by the National Institutes of Health. The protocol was approved by the Committee on the Ethics of Animal Experiments of the College of Gansu Traditional Chinese Medicine Experimental Animal Center (Permit Number: SYXK 2011–0001). All surgeries were performed under sodium pentobarbital anesthesia, and all efforts were made to minimize the suffering of the animals.

### Statistics

The values are presented as the mean ± standard error (SE). Statistical analyses were performed using SPSS 17.0 software (SPSS Inc., Chicago, IL, USA). Comparisons among the SCH, SCH+T4 and control groups were assessed by ANOVA. Vasorelaxation was calculated as the percent of maximal contraction following NE exposure. The responses to NE were compared by two-way ANOVA followed by the Bonferroni test to determine which comparisons were statistically significant. Pearson correlations were performed to determine the correlations between the various biomarkers and TSH. A p-value of less than 0.05 was considered to be statistically significant.

## Results

### Morphological variables

**Fig 1:**
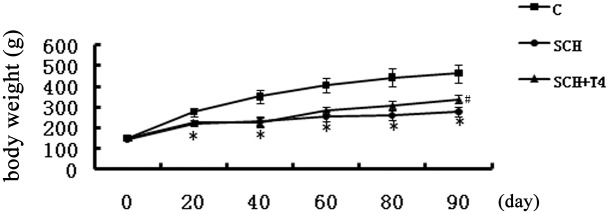
L-T4 treatment improved the body weights of SCH rats. The data are expressed as the mean±standard error (SE). *P<0.05 vs. control group.

**Fig 2:**
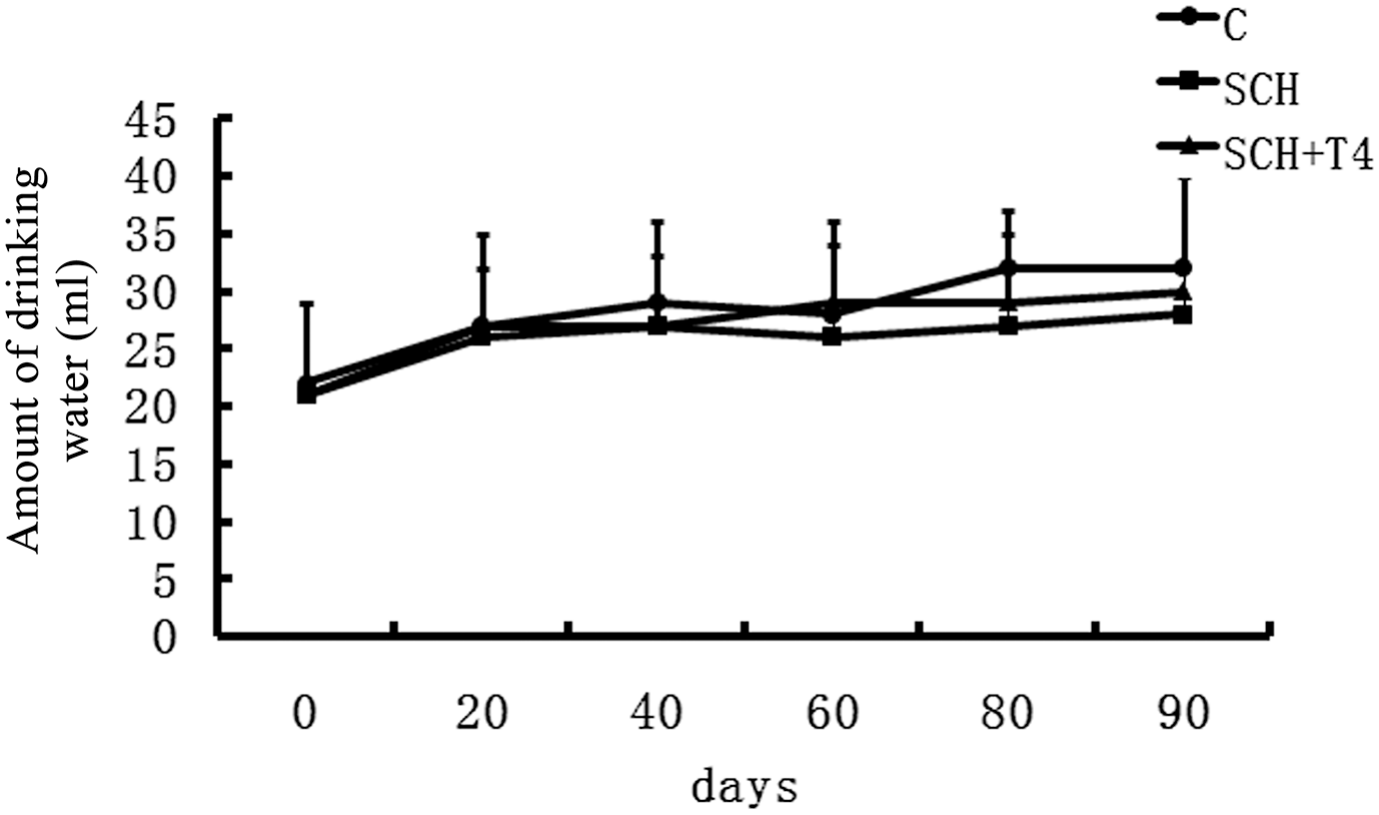
No significant difference was observed in water consumption among the three groups. The data are expressed as the mean±standard error (SE).

At the end of the 3-month study period, the SCH rats experienced a stunted growth pattern compared with the control rats. Upon gross inspection, the SCH rats seemed less active than the control rats and had dry fur. These results are the same as those of previous studies[33, 41, 42]. Body weights were improved in the SCH+T4 treatment groups (Fig 1). Upon gross inspection, physiological symptoms were also ameliorated in the SCH+T4 treatment groups. However, no significant difference was observed in water consumption among the three groups (Fig 2).

### Plasma thyroid hormone, TSH, lipid, NO and ET levels

The biochemical profiles of the three groups are shown in Table 1. SCH was confirmed by measuring plasma FT3, FT4 and TSH levels at three months after initiation of the experiment. The serum TSH concentration in the SCH group was significantly increased compared with that in the control group. Conversely, there was a decrease in its concentration in the L-T4 treatment group which approached that of the control group. The rats in the SCH group had higher serum TC and LDL-C levels compared with those of the rats in the control group (p<0.05). However, no significant differences in the HDL-C and TG levels were observed in the SCH group compared with the control and SCH+T4 groups. The SCH rats displayed an increased serum ET level and a decreased NO level compared with the control rats (p<0.05). No significant differences in the biochemical profiles were observed between the L-T4 group and the control group.

**Table 1:** Serum thyroid hormone, TSH, lipids, ET and NO levels.

	C	SCH	SCH+T4
TT3 (ng/ml)	0.87±0.15	0.81±0.12	0.83±0.19
TT4 (ng/ml)	59.82±9.56	52.31±10.3	54.12±9.43
TSH (μIU/ml)	0.95±0.48	17.4±6.41*	0.83±0.29^#^
TC (mmol/l)	1.26±0.06	2.38±0.55*	1.29±0.05^#^
LDL-C(mmol/l)	0.40±0.08	1.02±0.32*	0.42±0.04^#^
TG (mmol/l)	0.43±0.09	0.44±0.08	0.42±0.05
HDL-C(mmol/l)	0.78±0.04	0.80±0.02	0.79±0.03
ET (nmol/l)	93.41±17.23	160.62±37.25*	98.54±32.43^#^
NO (μmol/l)	314.75±32.69	235.13±20.14*	307.69±32.85^#^

Data are expressed as the mean±standard error (SE).

*P<0.05 vs. control group;

^#^P<0.05 vs.

SCH group; NS, not significant. TT3: Total triiodothyronin; TT4:Total thyroxin; TSH: thyroid-stimulating hormone; TC: Total cholesterol; LDL-C: Low-density lipoprotein cholesterol; TG: Triglyceride; HDL: high density lipoprotein cholesterol; ET: endothelin; NO: nitric oxide.

### Hemodynamic data

**Fig 3:**
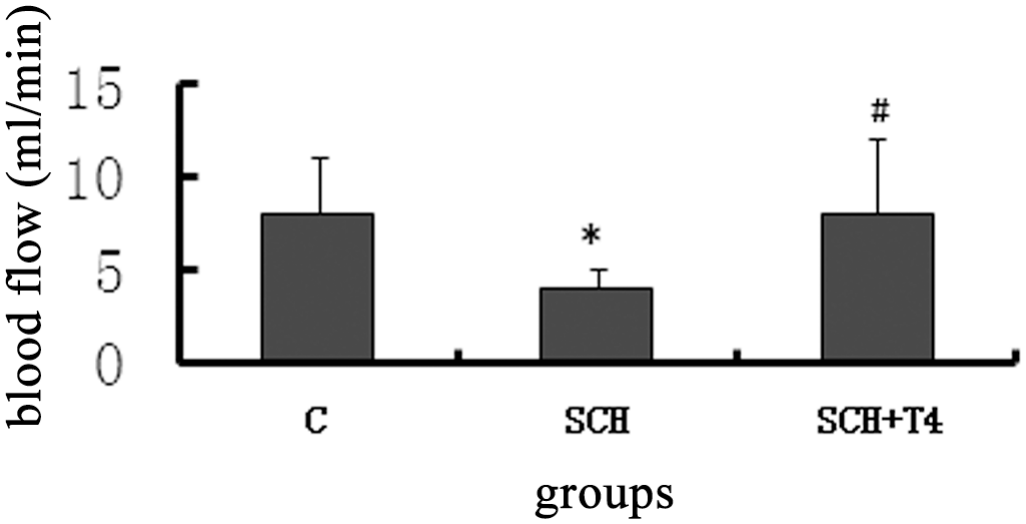
Aorta ventralis blood flow was decreased in the SCH group and was improved by L-T4 treatment. The data are expressed as the mean±standard error (SE). *P<0.05 vs. control group; ^#^P<0.05 vs. SCH group.

The carotid artery as well as the tail MBP, SBP and DBP values were decreased in the SCH rats compared with the control rats (p<0.05). The SCH+T4 group displayed an increase in BP after the L-T4 treatment compared with the SCH rats (p<0.05, Table 2). The hemodynamic data (Table 3) confirmed the deterioration of cardiac functioning in the SCH group rats. This was indicated by a 12% reduction in the LV systolic pressure, a 29% reduction in the maximal rate of pressure rise (dP/dt), and an 81% increase in the contractility index (1/s) compared with those of the control group rats (p<0.05). Each of these parameters was improved following T4 treatment. The left ventricular systolic pressure, mean pressure, heart rate, EDP and-Min dP/dt (mmHg/s) were decreased in the SCH rats and were improved in the SCH+T4 rats. The LV isovolumic relaxation time constant was markedly increased in the SCH rats compared with the control and SCH+T4 rats (p<0.05). As shown in Fig 3, the SCH rats displayed a significant decrease in aorta ventralis blood flow compared with the control rats. The L-T4 treatment rats had higher aorta ventralis blood flow compared with that of the SCH rats (p<0.05).

**Table 2:** Blood pressure.

		C	SCH	SCH+T4
Tail blood pressure	SBP(mmHg)	157±13	116±6*	165±11^#^
	MBP(mmHg)	136±15	99±6*	144±13^#^
	DBP(mmHg)	126±17	91±7*	134±15^#^
Carotid artery blood pressure	SBP(mmHg)	127±15	111±6*	122±10^#^
	MBP(mmHg)	121±13	98±7*	118±8^#^
	DBP(mmHg)	108±13	86±8*	101±8^#^

Data are expressed as the mean±standard error (SE).

*P<0.05 vs. control group;

^#^P<0.05 vs.

SCH group; SBP: systolic blood pressure; MBP: mean blood pressure; DBP: diastole blood pressure.

**Table 3:** Ventricular parameter.

	C	SCH	SCH+T4
LV Sys P(mmHg)	121±12	108±12*	123±5^#^
LVEDP (mmHg)	13±10	5.8±5*	18±7^#^
Mean Pressure (mmHg)	63±10	51±9*	65±4^#^
Heart Rate (BPM)	403±28	347±35*	397±51^#^
Min dP/dt (mmHg/s)	-7376±773	-4672±670*	-6445±361^#^
Contractility Index (1/s)	62±7	111±16*	61±17^#^
Max dP/dt (mmHg/s)	6507±831	4861±573*	6849±342^#^
Tau(s)	0.02±0.001	0.03±0.003*	0.0184±0.004^#^

Data are expressed as the mean±standard error (SE).

*P<0.05 vs. control group;

^#^P<0.05 vs.

SCH group; LV Sys P: left ventricular systolic pressure; LVEDP: left ventricular end-diastolic pressure; HR, heart rate; Min dP/dt: minimal rate of pressure decline. Max dP/dt: maximal rate of pressure rise; Tau, time constant of LV isovolumic relaxation; BMP, beats/min.

### Vasorelaxation responses

**Fig 4:**
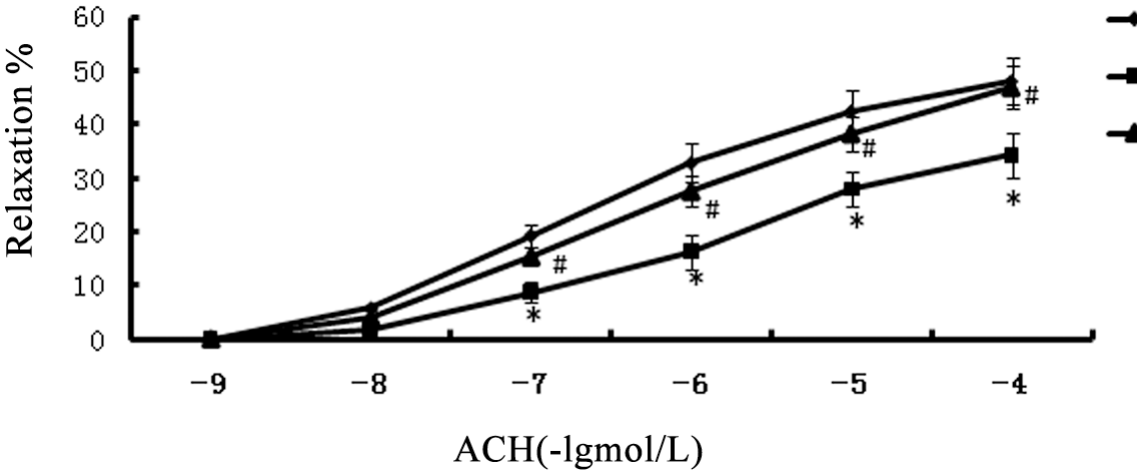
L-T4 treatment improved endothelium-dependent relaxation in SCH rats. Endothelium-dependent relaxation in response to acetylcholine in rat abdominal aorta. Experiments were performed using control, SCH and SCH+T4 group rats. The data are presented as the mean±SE of 20 experiments. *P<0.05 vs. control group; ^#^P<0.05 vs. SCH group.

Norepinephrine (NE) (10–9 to 10–4 M) caused endothelium-dependent contraction in the rats in all groups. The contractile responses to NE (10–7 M) administered to the precontracted vascular rings did not differ among the groups (data not shown). The ACH treatment caused concentration-dependent relaxation, which was lower in the SCH group arteries compared with the control arteries (p<0.05). However, the L-T4 treatment group had a higher rate of aorta ventralis blood flow compared with the SCH group (p<0.05, Fig 4).

### Histopathological analysis of the arcus aorta

**Fig 5:**
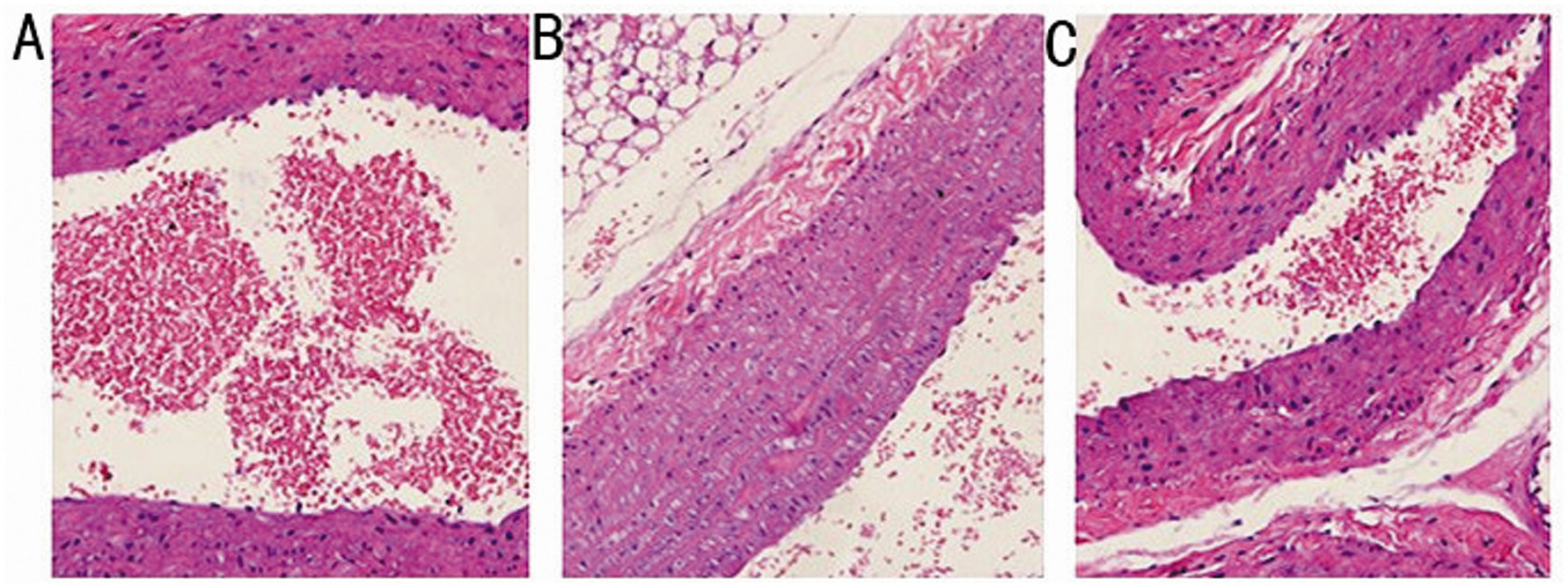
There is no significant pathological changes of the aortic arch in any of the three study groups. Representative histological sections of the arcus aortae wall stained with H&E (200x). A: SCH group; B:SCH+T4 group; and C: control group.

Histopathological examination revealed that the endothelial area displayed no pathological changes in the SCH rat arteries. There was no histopathological evidence of an irregular lumen, impairment or desquamation of endothelial cells, intimal thickening and fibrosis, or foam cell formation in the aorta due to inhibition of mononuclear cell adhesion (Fig 5).

### Pearson correlation analysis of various variables

Body weight is a confounder of the association of lipids and hemodynamics; thus, we set body weight as a control variable. Pearson correlation analysis revealed that the ET (p = 0.007, R = 0.785), contractility index (p = 0.039, R = 0.627), and Tau(s) (p = 0.001, R = 0.846) were positively correlated with the TSH level. Furthermore, the TSH level was negatively associated with the NO level (p = 0.01, R = -0.833), tail SBP (p = 0.003, R = -0.810), Max dP/dt (p = 0.042, R = -0.649), LV Sys P (p = 0.01, R = -0.733) and HR (p = 0.016, R = -0.704).

## Discussion

Thyroid disorders are common endocrine diseases. Several studies have demonstrated that SCH is a risk factor for coronary disease and atherosclerosis[43, 44], but other studies have not[21, 22]. The present study revealed that SCH altered serum lipid levels, blood pressure, cardiac function, aorta ventralis blood flow, endothelial functioning, and hemodynamics and that L-T4 treatment improved these levels.

SCH rats were induced by MMI administration (10 mg/kg) once daily by gavage. This dose was selected based on previous studies[30–32], and we reduced the dose by five-fold. After 45 days, a rat model of SCH was established. Our experiments were designed to assess the SCH rat model and to investigate the long-term effects of SCH on the heart. Therefore, we continued the MMI treatment and concurrently detected the thyroid hormone and TSH levels.

Endothelial dysfunction is the first step in coronary AS[45]. Evidence supporting the correlation between SCH and AS has been accumulating. And SCH may be related to endothelial dysfunction and AS in several ways[46, 47]. A recent study has revealed that SCH is associated with increases in carotid intima-media thickness and carotid plaque formation independent of classical AS risk factors [2]. However, a Danish study failed to find any association between SCH and CVD[48]. Endothelial dysfunction helps to predict cardiovascular events before they become apparent[13]. Similarly, endothelial dysfunction contributes to coronary artery disease progression and cardiovascular events[49]. Further, the endothelium maintains vascular function by producing vasodilator and vasoconstrictor substances. NO is the most important vasodilatory substance that is produced by the endothelium[50]. Therefore, decreased NO availability are associated with cardiovascular events[50]. Our study demonstrated that SCH rats displayed an increased serum ET level and a decreased NO level. Moreover, the vasodilation effect of acetylcholine was significantly reduced in the SCH rats, and it was increased after L-T4 treatment. We detected no significant pathological changes of the aortic arch in any of the three study groups. Because AS is a chronic disease, longer-term studies will be required to monitor histopathological changes[51].

Chronic hypothyroid patients display increased peripheral arterial resistance[52]. It is unclear whether SCH is associated with blood pressure alterations[53]. A recent meta-analysis conducted by Cai et al. has indicated that SCH is related to increases in SBP and DBP[54], but these results were not adjusted for age. Previous studies have indicated that SCH is not positively associated with hypertension or average arterial pressure[55], and they have not demonstrated that an increase in the serum TSH level to >10.0 mU/ml is associated with an elevation in blood pressure. In contrast, our study revealed that the SCH rats demonstrated decreased carotid artery and tail SBP, MBP and DBP. The treatment of these rats with L-T4 improved these parameters. Age and other confounding factors were controlled, and the serum TSH and lipid levels were not greatly increased in our study. Owen et al.[56] have observed that female subjects with SCH have a higher DBP and that L-T4 treatment reduces the BP of SCH patients. However, these subjects had much higher total cholesterol and LDL levels than the patients in our study.

The cardiovascular system is a specific target of thyroid hormones. Therefore, thyroid dysfunction is accompanied by profound changes in cardiovascular hemodynamics[57, 58]. A rise in the thyroid hormone level results in increases in myocardial contractility, relaxation, cardiac output, and heart rate, whereas hypothyroidism decreases these parameters[59, 60]. A previous study has revealed that patients with SCH have lower LV strain and strain rate values[61], however, the sample size was too small in this cross-sectional study. A study by Iqbal et al. included 66 subjects with SCH and did not find any significant differences in diastolic or systolic functioning compared with euthyroid controls[62]. Furthermore, in that study, the serum TSH levels in the SCH patients were only slightly above the reference range. The present study revealed that SCH leads to decreases in left ventricular systolic pressure (MAP), dP/dt, LV time constant and heart rate and an increase in the contractility index as well as that L-T4 treatment reverses these alterations. The results of the present study are consistent with the findings of a meta-analysis, suggesting that SCH worsens LV diastolic function parameters, with profound effects on cardiac structure and function[63].

L-T4 is the replacement therapy drug used in hypothyroidism. Taking the proper amount of L-T4 aids to increase serum FT4 and FT3 levels in SCH, and can reduce TSH secretion with negative feedback on the pituitary. Some studies have concurred that L-T4 improves endothelial functioning, left ventricular diastolic functioning and carotid intima-media thickness[64, 65]. The present study revealed that L-T4 treatment ameliorates endothelial dysfunction and hemodynamic changes in SCH rats. However, a negative study[66] has indicated that L-T4 treatment does not significantly improve endothelial functioning or reduce the carotid IMT in subjects with mild SCH of similar ages and BMIs and with similar smoking statuses, menopausal statuses, and endothelial function modifiers.

A previous study has indicated that the biologically active thyroid hormone T3 affects cardiac contractility, heart rate (HR), diastolic functioning and systemic vascular resistance through effects mediated by genomic and non-genomic factors[67]. However, the T3 levels in SCH rats were found to be normal. New data suggest that heart disease promotes a low cardiac T3 level, which may not be reflected in peripheral blood assays[68]. It is unclear whether these changes are associated with TSH. Pearson correlation analysis conducted in our study revealed that the ET, contractility index, and Tau(s) were positively correlated with the TSH level. In addition, the TSH level was negatively associated with the NO level, tail SBP, Max dP/dt, LV Sys P, and HR. The positive correlation between the serum TSH level and diastolic blood pressure may represent an increased cardiovascular risk. Previous studies have indicated that an elevated TSH level promotes vascular smooth muscle cells proliferation[69] and endothelial dysfunction[70] and that the TSH level is positively correlated with several cardiovascular risk factors[71]. Maintaining the serum TSH level within an appropriate range results in lipid-level homeostasis and slows AS progression [72, 73]. As the main characteristic of SCH, an elevation in the TSH level plays a key role in the development of AS. L-T4 treatment partially restores endothelial dysfunction and the hemodynamic changes resulting from a decreased serum TSH level. Therefore, we can speculate that a high TSH level plays an important role in the mechanism leading to these changes.

Our data demonstrate that L-T4 treatment is beneficial for restoring endothelial cells with early stage injury. Early identification of SCH may lead to early treatment, thereby favorably affecting the cardiovascular outcome. The current evidence should be confirmed in larger trials, and the impact of L-T4 on other cardiovascular risk factors or previous cardiovascular diseases needs to be explored. Further mechanisms should be investigated to explain the role of SCH in CV disease.

### Limitations

Our study has several limitations. First, although the pressure-volume methodology could be a very useful approach for the assessment of cardiac function in rats, the invasive detection can only be used in animals. Second, our investigation excluded female rats, which restricts our results. Third, the histopathological findings during our experiments suggest that the duration of the experiment was not sufficiently long.
